# Changes in Placentas of Pregnant Women Infected with COVID-19

**DOI:** 10.3390/ijms26178596

**Published:** 2025-09-04

**Authors:** Solomiia Kramar, Zoia Nebesna, Yuliia Yakymchuk, Alla Boychuk, Oksana Shevchuk, Mykhaylo Korda, Sandor George Vari

**Affiliations:** 1 Department of Histology and Embryology, I. Horbachevsky Ternopil National Medical University, 46001 Ternopil, Ukraine; nebesna_zm@tdmu.edu.ua; 2 Department of the Obstetrics and Gynecology, I. Horbachevsky Ternopil National Medical University, 46001 Ternopil, Ukraine; julli_yakumchuk@ukr.net (Y.Y.); boychuk_alla@tdmu.edu.ua (A.B.); 3 Department of Pharmacology and Clinical Pharmacology, I. Horbachevsky Ternopil National Medical University, 46001 Ternopil, Ukraine; shevchukoo@tdmu.edu.ua; 4 Department of Medical Biochemistry, I. Horbachevsky Ternopil National Medical University, 46001 Ternopil, Ukraine; korda@tdmu.edu.ua; 5 International Research and Innovation in Medicine Program, Cedars–Sinai Medical Center, Los Angeles, CA 90048, USA

**Keywords:** COVID-19, placenta, pathological changes, histopathology, ultrastructure

## Abstract

SARS-CoV-2 infection in pregnant women can lead to pregnancy-related complications. This work aims to study the spectrum of pathological changes in the placentas of SARS-CoV-2-infected pregnant women. The study involved 50 pregnant women with COVID-19 disease in the first (group I), second (group II), and third (group III) trimesters. Placental sections were examined by histopathology, electron microscopy, and immunohistochemistry to assess structural and molecular changes. The placentas of SARS-CoV-2-affected pregnant women exhibit nonspecific pathological changes, primarily associated with impaired blood circulation. The most frequent findings include thrombosis, chorangiosis, villous edema, and fibrinoid necrosis, all indicative of endothelial dysfunction. Increased expression of sclerostin and Annexin A2 was also detected in affected placentas. The main submicroscopic manifestations of placental insufficiency in COVID-19-affected women are dystrophic–destructive changes in the stroma of the villi, manifested by edema and fibrous processes, which cause significant disruption of the fetoplacental barrier. SARS-CoV-2 causes thrombotic and sclerotic changes, mainly in the maternal portion of the placenta. The manifestation of pathological changes in the placenta of COVID-19-affected women depends on the pregnancy period during which infection by SARS-CoV-2 has occurred. The established findings may provide insights into the connection between COVID-19 in pregnancy and antenatal and perinatal outcomes.

## 1. Introduction

At the end of 2019, severe acute respiratory syndrome coronavirus 2 (SARS-CoV-2) was initially detected in Wuhan, Hubei Province, China. This novel coronavirus quickly spread globally, leading to a pandemic. The illness it causes is known as coronavirus disease 2019 (COVID-19) [1,2,3,4]. The total number of COVID-19 deaths reported to WHO at the end of 2024 was 7.1 million [5].

COVID-19 can cause damage to various organs, including the lungs, heart, kidneys, liver, blood vessels, and others. Respiratory failure and acute respiratory distress syndrome (ARDS) are among the most frequent complications associated with severe COVID-19 infection [6]. A variety of potential late complications after COVID-19 infection include lung fibrosis, arterial thromboses, venous thromboembolism (VTE), cardiac thrombosis and inflammation, stroke, “brain fog,” dermatological issues, and overall mood dysfunctions [6,7]. COVID-19 is also associated with an increased risk of an unfavorable pregnancy. Results showed that pregnant women infected with SARS-CoV-2 had a higher risk of maternal death, fetal death/stillbirth, preterm birth, preeclampsia, and cesarean delivery [8,9,10]. According to a sizeable Nordic research study that included 389,949 births across Norway, Sweden, and Denmark, there is a 2.4 times higher risk of stillbirth among women infected with COVID-19 during pregnancy compared to those who were not infected [11]. Among 1,249,634 delivery hospitalizations in the USA during March 2020–September 2021, the stillbirth rate was 0.64% in pregnancies without COVID-19 and 1.26% in those with the infection [12].

The placenta serves as a critical source of clinically relevant information. Examining placental tissue provides valuable insights into fetal development, neonatal morbidity and mortality, and the future health of both the mother and the fetus [13,14,15]. Besides the direct effect of SARS-CoV-2 through vertical transmission, the fetus can be indirectly affected by placental lesions that impair placental villous function [16]. Numerous scientific publications have already demonstrated various pathological changes at the microscopic level in the placentas of women with SARS-CoV-2 infection [4,14,17,18,19,20,21,22,23,24,25]. They lead to systemic vasculopathy and inflammatory processes in the organ [16,21,26].

Several immunohistochemical studies in this area have focused on detecting SARS-CoV-2 spike protein, SARS-CoV-2 nucleocapsid, and proinflammatory cells [19,21,23,24,25,27,28,29], while electron microscopic studies have concentrated on detecting virions [4,27,29].

Our work aims to focus on the study of the morphological restructuring of the placenta under the influence of COVID-19 infection in different trimesters of pregnancy.

## 2. Results

**Figure 1:**
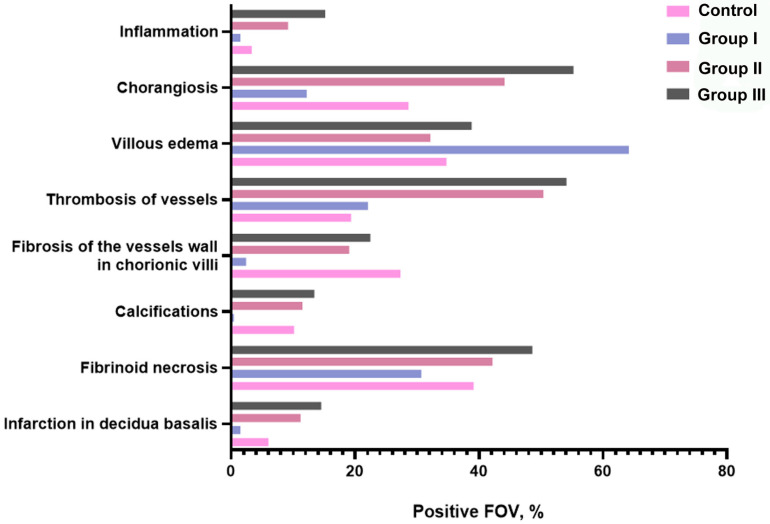
Distribution of placental microscopic findings (percentages) in different groups.

Microscopic examination of the placenta of women of different groups revealed the following pathological changes: infarction in decidua basalis, fibrinoid necrosis, calcifications, fibrosis of the vessels wall in chorionic villi, thrombosis of vessels, villous edema, chorangiosis, and inflammation. Most thrombotic and sclerotic lesions resulting from COVID-19 infection were found in the maternal portion of the placenta (Figure 1).

**Figure 2:**
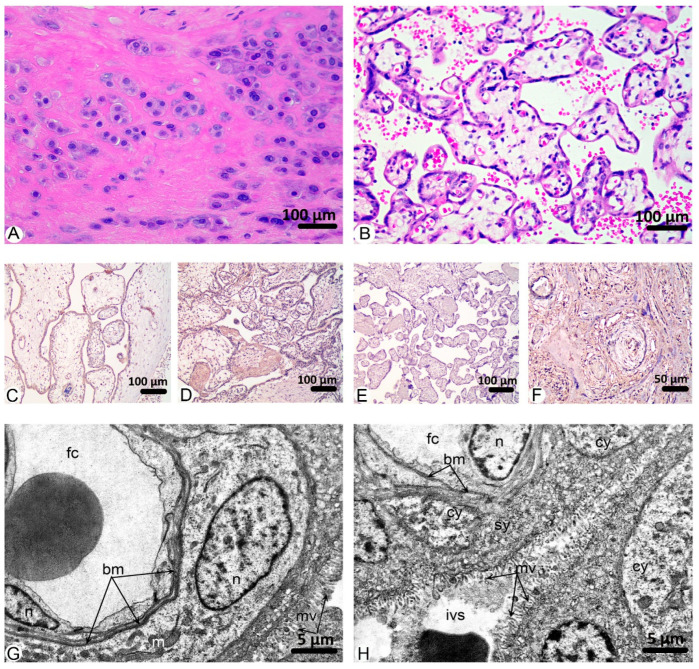
Findings in the placentas of the control group. ***Microscopic images (H&E):*** (**A**)**.** Groups of decidual cells in the decidua basalis. (**B**)**.** Tertiary chorionic villi. ***Immunohistochemistry:*** (**C**)**.** VEGF A. Modern staining intensity in syncytio-, cytotrophoblast and endothelium of villi vessels. (**D**)**.** PlGF. Moderate staining intensity in syncytio-, cytotrophoblast, and fibrinoid necrosis areas. (**E**)**.** Sclerostin. Weak staining intensity in chorionic villi. (**F**)**.** Annexin A2. Moderate staining intensity in placental structural components. ***Transmission electron microscopy:*** (**G**)**.** Hematoplacental barrier. Hemocapillary trophoblast villi (fc), a typical structure of a cytotrophoblast cell with a euchromatin nucleus (n), in the cytoplasm there are elongated mitochondria with a relatively electron-dense matrix (m), syncytiocapillary basement membrane (bm), a fragment of an endotheliocyte nucleus (n), trophoblast microvilli (mv). (**H**)**.** Hematoplacental barrier. Hemocapillary trophoblast villus (fc), endotheliocyte nucleus (n), syncytiotrophoblast (sy), cytotrophoblast cytoplasm fragment (cy), hemocapillary basement membrane (bm), trophoblast microvilli (mv), intervillous space (ivs).

Histological analysis of the control group’s placental sections revealed the preservation of its structural components, including the decidua basalis, blood-placental barrier, chorionic plate, and amniotic epithelium (Figure 2A,B). Among the pathological changes that we noticed, the most significant percentage of fields of view was occupied by fibrinoid necrosis, 39.11%, and villous edema, 34.74% (Figure 1).

Immunohistochemical analysis of the control placenta samples revealed the moderate intensity of immunostaining against VEGF A, PlGF, Annexin A2, and weak for sclerostin (Figure 2C–F).

Ultrastructural studies of the placenta of women in the control group showed a typical structure. A thin layer of syncytiotrophoblast forms the blood placental barrier, individual cytotrophoblast cells are located on a thin basement membrane, which in certain areas merges with the basement membrane of hemocapillaries of placental villi, and a standard syncytiocapillary basement membrane is formed. Numerous microvilli are found on the surface of the syncytiotrophoblast directed into the intervillous space. The wall of the hemocapillaries in placental villi is formed by endothelial cells, which contain a nuclear organelle zone and a thin, peripheral zone comprising micropinocytotic vesicles and caveolae. Microvilli are present on the lumenal surface. Endotheliocytes are located on a continuous basement membrane, in the splits of which there are pericytes on the outside. The stroma of the villi is formed by loose connective tissue with a moderate content of leukocytes. In the intervillous space, formed elements of the maternal blood are present, mainly erythrocytes (Figure 2G,H).

**Figure 3:**
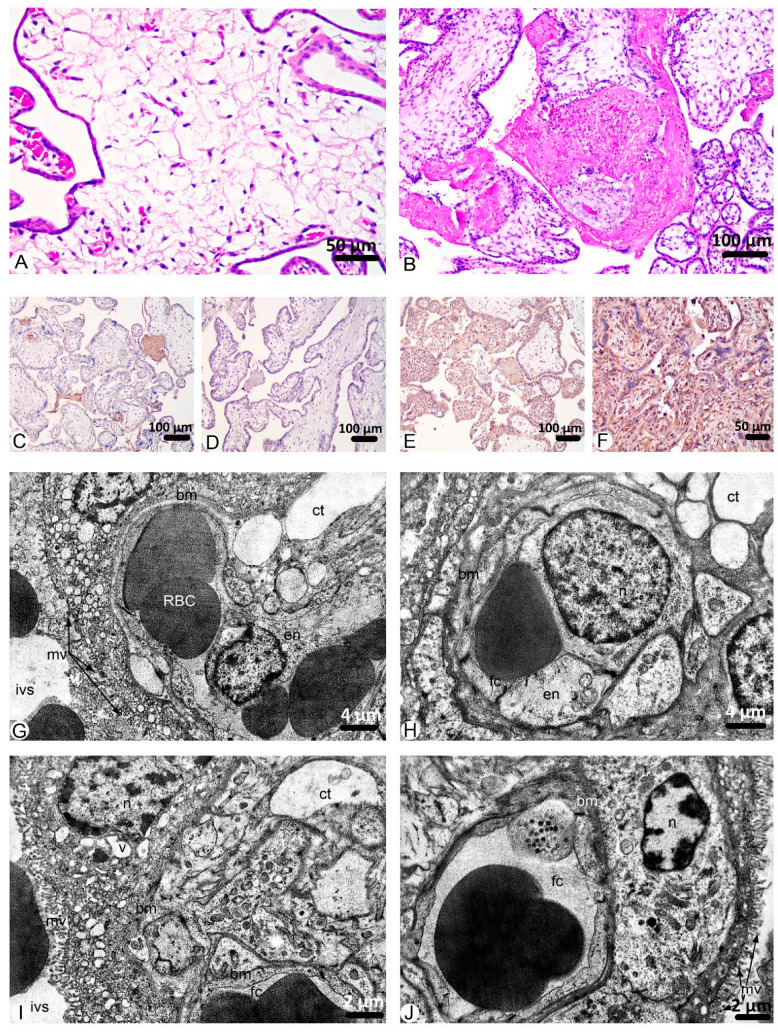
Findings in the placentas of patients who were diagnosed with COVID-19 during the 1st trimester of pregnancy. ***Microscopic images (H&E):*** (**A**)**.** Villous edema. (**B**)**.** Fibrinoid necrosis. ***Immunohistochemistry:*** (**C**)**.** VEGF A. Weak cytoplasmic and moderate serum immunostaining. (**D**)**.** PlGF. Weak immunostaining. (**E**)**.** Sclerostin. Moderate immunostaining. (**F**)**.** Annexin A2. Strong immunostaining. ***Transmission electron microscopy:*** (**G**)**.** Ultrastructural changes of the placental villi. Destruction and swelling of the villous stroma (ct), nucleus and swollen homogeneous endotheliocyte cytoplasm (en), basement membrane (bm), aggregation of formed blood elements in the capillary lumen of the trophoblast (RBC), trophoblast, low fragmented microvilli (mv), intervillous space (ivs). (**H**)**.** Ultrastructural changes in hemocapillary and villous stroma. Swelling of the loose connective tissue of the villous stroma (ct), the nucleus of an endotheliocyte (n), the swollen cytoplasm of an endotheliocyte (en), a narrow lumen of a capillary with an erythrocyte (fc), a deformed basal membrane of a capillary (bm). (**I**)**.** Ultrastructural changes in the villi. Edema of the loose connective tissue of the villous stroma (ct), capillary lumen with erythrocytes (fc), capillary basement membrane (bm), troblast nucleus (n), small, numerous vacuoles in the trophoblast cytoplasm (v), indistinct, homogeneous trophoblast basement membrane (bm), microvilli (mv), intervillous space (ivs). (**J**)**.** Changes in the hematoplacental barrier. Capillary lumen with erythrocytes (fc), indistinct syncytiocapillary basement membrane (bm), cytothrothroblast nucleus (n), partially reduced microvilli (mv).

Placenta morphology of group I under microscopic research shows a high percentage of villous edema (64.14%) (Figure 3A) mainly observed in secondary villi. Other the most frequent changes in this group are fibrinoid necrosis (30.67%) (Figure 3B) and thrombosis of vessels (22.06%). Only 12.19% of FOV were with chorangiosis in the terminal villi. Signs of other pathological findings were observed in a few FOV (Figure 1).

Through immunohistochemical research of group I placenta samples, weak cytoplasmic and moderate serum immunostaining for VEGF A, a weak reaction for PlGF, a moderate reaction for sclerostin, and a strong reaction for Annexin A2 were identified (Figure 3C–F).

An ultrastructural study of the placentas villi of group I of women diagnosed with COVID-19 during the 1st trimester of pregnancy showed destructive changes in the stroma and components of the blood placenta barrier. In this group, significant swelling of the stromal loose connective tissue of placental villi was established, and thin collagen fibrils, fragmented or compacted in some areas, were detected in the electron light matrix. Among the cellular components, mainly fibroblasts containing a large, round-oval euchromatin nucleus and cytoplasm, in which organelles of the synthetic apparatus are well developed: endoplasmic reticulum and Golgi complex were identified.

Hemocapillaries of the villi were full of blood with phenomena of stasis and sludge; the lumens were mainly narrowed, but they were moderate in some microvessels. The cytoplasm of endotheliocytes was also swollen, but the integrity of membrane organelles was preserved. The integrity of intercellular contacts is not violated. Basal membrane without pronounced alterative changes, mostly homogeneous with high electron density.

Villi are mainly covered with cytotrophoblast; since the placentation period is not yet complete, syncytiotrophoblast is rarely detected. The basement membrane of the trophoblast or typical syncytiocapillary basement membrane was moderately thick, locally thickened. Trophoblast cells contained oval-shaped nuclei of moderate electron density, where euchromatin prevailed, and nucleoli were determined. The cytoplasm contained well-developed, moderately dilated tubules and vacuoles of endoplasmic reticulum and Golgi complex cisternae. Glycogen inclusions and destructed mitochondria with an electron-bright matrix and partially fragmented cristae were revealed. Syncytiotroblast nuclei contained mainly heterochromatin and had an irregular shape; most organelles were destructed in the cytoplasm, and numerous small vacuoles were found. The apical surface of the trophoblast contained microvilli, but they were short, fragmented, or reduced; however, in most fields of view, they were unchanged (Figure 3G–J).

**Figure 4:**
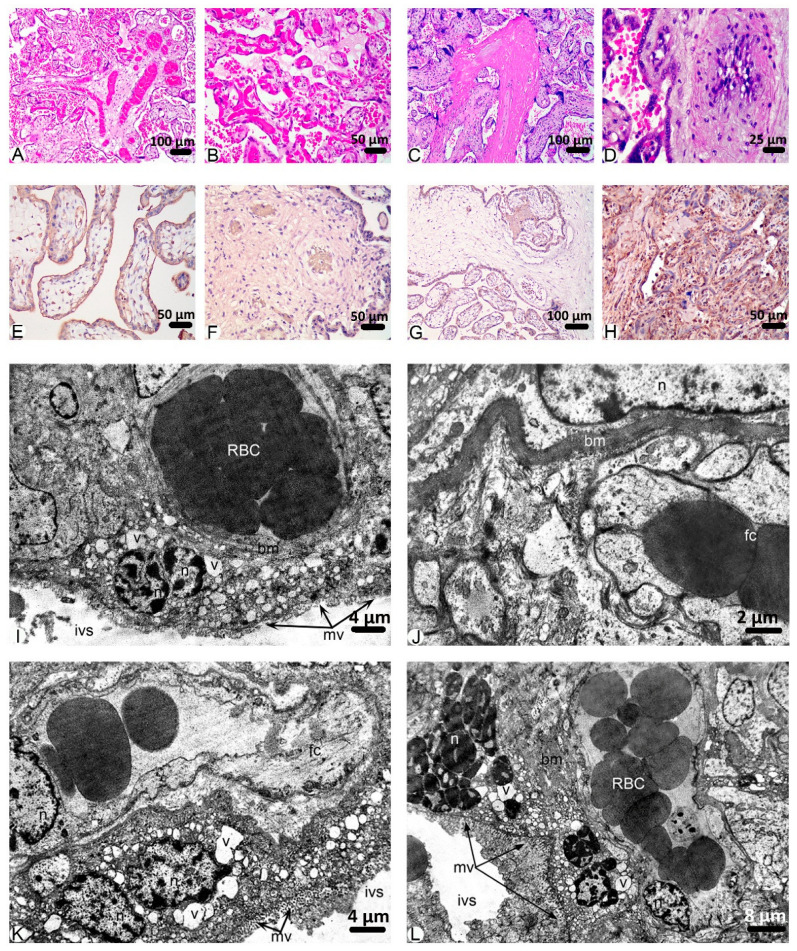
Findings in placentas of patients who were diagnosed with COVID-19 during the 2nd trimester of pregnancy. ***Microscopic images (H&E):*** (**A**)**.** Thrombosis of vessels. (**B**)**.** Chorangiosis. (**C**)**.** Fibrinoid necrosis. (**D**)**.** Fibrosis of the vessel wall. ***Immunohistochemistry:*** (**E**)**.** VEGF A. Moderate immunostaining. (**F**)**.** PlGF. Moderate immunostaining. (**G**)**.** Sclerostin. Moderate immunostaining. (**H**)**.** Annexin A2. Strong immunostaining. ***Transmission electron microscopy:*** (**I**)**.** Changes in the hematoplacental barrier. Stasis of erythrocytes in the lumen of the fetal capillary (RBC), syncytiocapillary basement membrane (bm), vacuoles in the cytoplasm of the trophoblast (v), apoptotic nuclei of the trophoblast (n), disorganized and fragmented microvilli (mv), intervillous space (ivs). (**J**)**.** Changes in the hematoplacental barrier. Swelling of the endothelial cell’s cytoplasm of the capillary of the trophoblast villus (fc), thickened basal membrane of the trophoblast (bm), a large nucleus of the trophoblast (n) with finely dispersed chromatin. (**K**)**.** Changes in the hematoplacental barrier. Swelling and damages of the endothelial cell’s cytoplasm (fc), the nucleus of the endotheliocyte (n), nuclei of the syncytiotrophoblast (n), vacuoles (v) in the syncytiotrophoblast’s cytoplasm, short microvilli (mv), the intervillous space (ivs). (**L**)**.** Changes in the hematoplacental barrier. Erythrocyte aggregation with “sludge” phenomenon in the capillary lumen (RBC), endotheliocyte nucleus (n), homogeneous syncytiocapillary basement membrane (bm), syncytiotrophoblast apoptotic nuclei (n) in syncytial buds, vacuoles in syncytiotrophoblast cytoplasm (v), fragmented microvilli (mv), intervillous space (ivs).

In the second group, the most abundant pathology was thrombosis of blood vessels, which was observed in 50.37% FOV (Figure 4A). A high percentage of FOV with chorangiosis (44.12%) (Figure 4B) and fibrinoid necrosis (42.16%) (Figure 4C) also were revealed. Villous edema and fibrosis of vessel walls (Figure 4D) are frequently observed in the placenta samples of this group (32.15% and 19.05%, respectively) (Figure 1 and Figure 4).

Moderate intensity reactions against VEGF A, PlGF, and sclerostin were detected with the immunohistochemistry method in the placenta samples of patients who were diagnosed with COVID-19 during the 2nd trimester of pregnancy. A strong reaction with an antibody against Annexin A2 was observed in the placenta structural components of the second group (Figure 4E–H).

Submicroscopic studies of the women placentas of group II showed significant alterative changes in all their structural components in the terminal (trophic) villi. In the swollen stroma of the villi, there are destructively changed vessels. Compensatory expanded lumens of hemocapillaries and venules are excessively filled with blood, and stasis and sludge effects of erythrocytes are revealed. Intravascular erythrocyte thrombi are found in some microvessels. The cytoplasm of endotheliocytes is bright and swollen and contains destructively changed organelles, single micropinocytotic vesicles, and caveolae. There is damage to the lumenal surface of the plasmalemma. Irregularly shaped nuclei with karyolemma intussusception, karyoplasm contained large lumps of marginally located heterochromatin. Intercellular contacts are preserved. The basal membrane of the hemocapillaries is thickened and contains thickened and thin areas, mostly indistinct and homogeneous.

The trophoblast in the composition of the chorionic villi is located on the basal membrane, which is mainly thickened and homogeneous; only sometimes are there areas of its compaction. Cytrophoblast cells with rounded-oval nuclei, in which euchromatin prevails, are located on the basal membrane. The karyolemma has indistinct membrane contours and few nuclear pores. The cytoplasm also exhibits weak electron density and contains organelles that are destroyed. The syncytiotrophoblast is characterized by atypical thickening. Numerous vacuole-like structures are identified in the cytoplasm. The nuclei form osmiophilic, even multi-layered clusters (syncytial buds), and they undergo apoptotic changes, appearing intensely osmiophilic. Microvilli on the apical surface are low and fragmented. The intervillous space contains formed elements of blood, mainly erythrocytes, and fibrinoid accumulation (Figure 4I–L).

**Figure 5:**
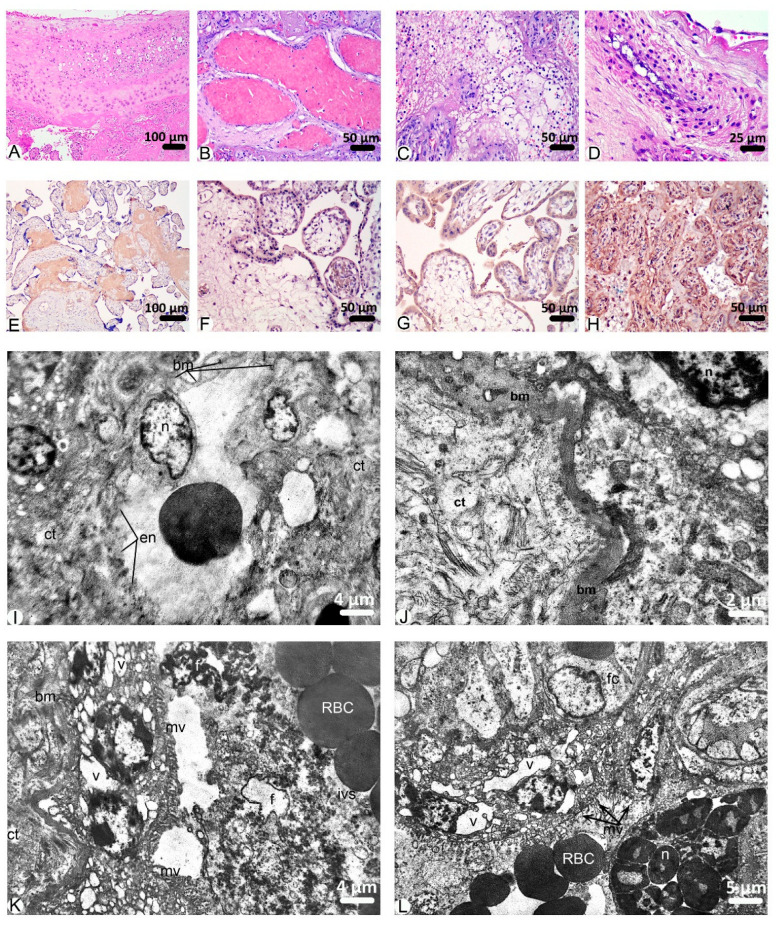
Findings in placentas of patients who were diagnosed with COVID-19 during the 3rd trimester of pregnancy. ***Microscopic images (H&E):*** (**A**)**.** Infarction in decidua basalis. (**B**)**.** Thrombosis of vessels. (**C**)**.** Inflammation. (**D**)**.** Fibrosis of the vessel wall. ***Immunohistochemistry:* (E).** VEGF A. Moderate immunostaining. (**F**)**.** PlGF. Moderate immunostaining. (**G**)**.** Sclerostin. Moderate immunostaining. (**H**)**.** Annexin A2. Strong immunostaining. ***Transmission electron microscopy:*** (**I**)**.** Ultrastructural changes of the placental villous capillary. Endotheliocyte nucleus (n) protruding into the hemocapillary lumen, damaged endotheliocyte cytoplasm (en), and damaged basement membrane (bm), which is practically not visualized, some of its fragments are present; perivascular, destructured connective tissue (ct). (**J**)**.** Villous stroma destruction (ct), thick trophoblast basement membrane (bm), destructured trophoblast cytoplasm with pyknotic osmiophilic nucleus (n). (**K**)**.** Changes in the hematoplacental barrier. Destruction of the villous stroma (ct), homogeneous fragmented villous basement membrane (bm), apoptotic trophoblast nuclei (n), vacuoles in the trophoblast cytoplasm (v), destructured microvilli (mv), fibrinoid deposits (f) in the intervillous space (ivs), lacunar blood (RBC). (**L**)**.** Changes in the hematoplacental barrier. Hemocapillaries of villi with swollen endotheliocytes (fc), deformed basement membrane (bm), vacuoles in the trophoblast cytoplasm (v), apoptotic nuclei of syncytiotrophoblast (syncytial bud) (n), damaged and deformed microvilli (mv), intervillous space (ivs) with red blood cells (RBC).

In the third group, pathological findings in the placenta were more frequently observed than in the previous groups. The majority of FOV contains thrombosis of vessels (54.09%), chorangiosis (55.23%), and fibrinoid necrosis (48.59%). Villous edema and fibrosis of vessels are widely spread pathologies in this group as well (38.81% and 22.45%, respectively) (Figure 1 and Figure 5).

The results of the immunohistochemical analysis of the placentas from the third group were identical to those in the second group. Reactions against VEGF A, PlGF, and sclerostin occurred with moderate intensity, while those with Annexin A2 occurred with strong intensity (Figure 5E–H).

Submicroscopic studies of the placentas of women in group III established deep destructive-degenerative changes in all structural components of the terminal villi. In the stroma, there was moderate swelling, coarse bundles of collagen fibers fragmented in many areas, and cell infiltrates of the histiocytic and leukocyte series were determined.

As in the previous observation group, the hemocapillaries of the villi are expanded, filled with blood, sludge, and thrombi, with erythrocytes being the main cell type found within them. Their walls suffered significant damage to the ultrastructure. Endothelial cells were characterized by swelling of the cytoplasm, reduction in micropinocytic vesicles, damage to the integrity of organelles, and their swelling, but intercellular contacts were preserved. A characteristic feature of the basal membrane of the hemocapillaries and over a longer length of the ordinary and villous placenta, on which the trophoblast is located, is its significant thickening or densification on large areas and, in some places, homogenization. The cellular component cytotrophoblast represents the trophoblast—the nuclei of such cells contain electron-bright karyo- and cytoplasm, and syncytiotrophoblast, which includes large electron-dense, apoptotic multinucleated conglomerates that form syncytial buds. The syncytiotrophoblast is destructively altered and contains nuclei of increased osmiophilicity, in which electron-dense heterochromatin prevails. The karyolemma of the nuclei is indistinct and contains significant intussusceptions; the perinuclear space is locally expanded, forming significant electron-bright zones. This ultrastructure of the nuclei indicates their low functional state. The cytoplasm of the syncytiotrophoblast contains cytoplasm in which the density of organelles is low. Expanded tubules of the granular endoplasmic reticulum are revealed, which sometimes form large vacuole-like structures. Few ribosomes are found on their membranes. Individually vacuolated mitochondria with light matrix and reduced cristae are identified. Destructively changed, fragmented microvilli are observed on the surface of the syncytiotrophoblast. In the intervillous space, there are erythrocytes and structureless, osmiophilic conglomerates of fibrinoid (Figure 5I–L).

## 3. Discussion

The placenta is a fundamental organ for fetal development and growth. Any abnormalities can disrupt its functions and thus change the course of pregnancy and affect the condition of the fetus and the health of the child after birth. The placenta plays a protective role, preventing the penetration of many foreign antigens from the mother to the fetus due to the placental barrier, the main components of which are the trophoblast and Hofbauer cells (macrophages) [4,26,29].

In our work, we focused on the morphological remodeling of the placenta under the influence of coronavirus infection in different trimesters of pregnancy. It should be noted that the pathologies observed in the studied fields of view were found in all study groups, but in different percentages. The dominant aberrations in the control group and the group where women had an infection in the first trimester of pregnancy were fibrinoid necrosis and villous edema. It is worth noting that villous edema was detected 1.85 times more often in the first study group than in the control group. Vascular thrombosis, chorangiosis, and fibrinoid necrosis prevailed in the placentas of women with COVID-19 infection in the second trimester of pregnancy. Vascular thrombosis in the studied fields of view of the placentas of the second group was observed to be 2.60 and 2.28 times more frequent compared to the control and group I, respectively. Additionally, in the placentas of women in the second group (second trimester), infarction in the decidua basalis occurred 1.86 times more frequently than in the control group. At the same time, inflammation was observed 2.76 times more frequently. The largest percentage of pathologies in the third group were chorangiosis, vascular thrombosis, and fibrinoid necrosis. Compared to the other groups, the third study group (third trimester) was the one in which infarction in the decidua basalis and inflammation reached their maximum values. The detection of these pathologies allows us to conclude that these aberrations are nonspecific, as each of them occurs in both the placentas of women without COVID-19 infection during pregnancy and in those who were infected. However, the analysis of these comparisons and the percentages of pathomorphological changes indicates that the frequency of vascular alterations and inflammatory and necrotic processes increases significantly in the presence of SARS-CoV-2 infection. A growing body of recent scientific evidence demonstrates similar pathological placental lesions linked to COVID-19 infection. In particular, the following aberrations were found, namely, vascular alterations, inflammation, thrombo-embolic complications, ischemia and necrosis [14,17,18,19,20,21,23,24,25,29,30,31,32,33]. Previously, a team of authors led by Vari et al. (2023), having analyzed scientific publications, concluded that pathological changes and mechanisms of placental dysfunction under the influence of SARS-CoV-2 infection, preeclampsia, and gestational diabetes are typical [34].

Among the numerous studies that have highlighted pathomorphologic changes in the placenta, only a few have utilized electron microscopy to identify organ damage at the ultrastructural level. Utmost focused on detecting viral particles in placental components [4,27,29]. However, the article by Parcial et al. (2022) described the ultrastructure of three samples of SARS-CoV-2-infected placentas [29]. The presence of pycnotic, osmophilic nuclei in the villi wall, apoptotic bodies, depletion of trophoblast cell cytoplasm into organelles, and swelling of the villi are the alterations that our team of authors observed during the analysis of the experimental samples. Additionally, according to our results, the primary submicroscopic manifestations of placental insufficiency in COVID-19-affected women include dystrophic and destructive changes in the villous stroma, characterized by edema and fibrotic processes, leading to significant disruptions in the ultrastructure of the fetoplacental barrier components.

Immunohistochemical detection of VEGF A, PlGF, and AnxA2 expression in the placenta was performed to evaluate the placental angiogenesis. VEGF-A is primarily expressed in syncytiotrophoblasts, cytotrophoblasts, and stromal cells of placental villi and promotes the growth of blood vessels in the placenta, supporting the rapidly expanding network required during pregnancy [35,36]. VEGF-A expression is higher during the first trimester when angiogenesis is most active [37]. Its levels decrease as pregnancy progresses, and the placental vascular network matures [35]. We established moderate immunostaining against VEGF-A in the control, second, and third groups and weak immunostaining in the placentas of women infected with SARS-CoV-2 during the first trimester of pregnancy. From research by Yazihan et al. (2021) [38], we know that VEGF-A may not play a central role in the pathophysiology of adverse pregnancy outcomes related to COVID-19. However, SARS-CoV-2 infection may influence VEGF-A expression in the placenta, potentially exacerbating vascular dysfunctions through inflammatory and hypoxic pathways [38]. Previously, Rana et al. (2022) [39] also established that reduced VEGF-A activity has been implicated in preeclampsia, as insufficient angiogenesis leads to abnormal placental development [39].

PlGF, a member of the VEGF family, shares three-dimensional structural similarities with VEGF-A but exclusively binds to the VEGFR-1 receptor. PlGF is highly expressed in the placenta throughout all stages of gestation, and it is thought to regulate trophoblast growth and differentiation, highlighting its role during the invasion of trophoblasts into the maternal decidua [40]. Altered PlGF levels have been linked to conditions such as eclampsia and preeclampsia, with the latter being a known complication of gestational diabetes mellitus (GDM). Given the shared pathological changes observed in the placenta during GDM and preeclampsia, it has been hypothesized that PlGF might also play a role in the development and pathology of the placenta in GDM [41,42,43]. Smadja et al. (2021) [44] suggested that PlGF serves as a predictive biomarker for in-hospital mortality in COVID-19 patients and PlGF-blocking strategies could represent a novel therapeutic approach [44]. Furthermore, Kosinska-Kaczynska et al. (2023) [45] found that pregnant women with COVID-19 exhibit elevated sFlt-1/PlGF ratios compared to healthy pregnancies, with these increases being more pronounced in severe COVID-19 cases and those with hypertensive disorders. This imbalance between angiogenic and anti-angiogenic factors may contribute to placental dysfunction [45]. Additionally, increased PlGF levels have been implicated in the pathophysiology of COVID-19, potentially influencing leukocyte infiltration and angiogenesis [46]. Our findings indicate that the expression profile of PlGF closely mirrors that of VEGF.

We established moderate immunostaining of AnxA2 expression in the placentas of women without COVID-19 infection in anamnesis during pregnancy and women who were infected in the first trimester of pregnancy, while immunohistochemical analysis of the placentas of the second and third groups showed a strong expression signal. Annexins are naturally produced proteins with key roles in reducing inflammation, preventing cell death, and maintaining anticoagulant processes [47]. AnxA2, also referred to as chromobindin-8, lipocortin II, or placental anticoagulant protein, is found in various cell types, including endothelial cells, monocytes, macrophages, dendritic cells, epithelial cells, and trophoblasts [48]. In humans, elevated AnxA2 expression in acute promyelocytic leukemia is linked to excessive fibrinolysis and bleeding [49]. Conversely, reduced AnxA2 levels disrupt cell surface fibrinolysis, increasing the risk of venous thromboembolism [50]. The identification of anti-AnxA2 antibodies in COVID-19 patients highlights its potential involvement in the pathogenesis of SARS-CoV-2 and related post-infection complications [51].

Sclerostin (SOST), encoded by the SOST gene, is expressed in various organs, including bone, cartilage, liver, pancreas, kidney, heart, fetal skin, and placenta [52,53,54]. Recent studies have highlighted significant interest in the role of sclerostin in various pathological conditions. Clinical and genetic research suggests that inhibiting sclerostin may increase the risk of cardiovascular diseases, as sclerostin produced in arterial tissue is thought to provide a protective mechanism against atherosclerosis [55]. Elevated serum sclerostin levels observed in chronic kidney disease may result from several factors, including renal retention of sclerostin. However, it has also been reported that urinary sclerostin excretion rises as estimated glomerular filtration rates decline, potentially coupled with increased production by bone cells [56].

Papadopoulou et al. (2023) [57] demonstrated that placental sclerostin and LRP5 levels are overexpressed in pregnancies affected by GDM compared to non-GDM pregnancies. Additionally, the positive correlation between placental sclerostin levels, pregestational maternal body mass index, and fasting glucose concentrations suggests an adaptive mechanism in response to maternal hyperglycemia [57]. Direct evidence linking placental sclerostin expression to SARS-CoV-2 infection remains absent. According to our immunohistochemical analysis, COVID-19 infection appears to increase sclerostin expression in the placenta.

Kerschan-Schindl et al. (2023) [58] reported significantly higher serum levels of sclerostin and dickkopf-1 in SARS-CoV-2-infected patients compared to matched controls [58]. Similarly, Diaz-Castro et al. (2024) [59] observed higher levels of dickkopf-1in placental samples from mothers with COVID-19 compared to controls. Colostrum samples from mothers affected by COVID-19 showed elevated levels of sclerostin [59].

## 4. Materials and Methods

### 4.1. Patients

The study involved 50 pregnant women treated in Ternopil Municipal Hospital No. 2 between November 2020 and January 2022. Forty patients with a history of COVID-19, confirmed by PCR test, were divided into three groups, and 10 pregnant COVID-19-negative women served as a control group (Table 1).

The study design was approved by the I. Horbachevsky Ternopil National Medical University Ethical Committee (protocol number 61, dated 13 November 2020), which was conducted in accordance with the World Medical Association Declaration of Helsinki.

All participants provided written informed consent after being informed about the study’s objectives, design, and methods. Patient anonymity was strictly maintained.

Control group inclusion criteria were a physiological course of pregnancy and the absence of SARS-CoV-2–specific IgA, IgM, and IgG antibodies.

Inclusion criteria for Groups I–III were: PCR-confirmed SARS-CoV-2 infection during the first, second, or third trimester of pregnancy; positive IgA, IgM, or IgG serum antibodies to SARS-CoV-2; maternal age 18–45 years; body mass index (BMI) 18.5–29.9 kg/m^2^; singleton pregnancy; absence of severe somatic or psychiatric disorders; conception within 3–6 months before enrollment; and consent to undergo additional diagnostic procedures.

Exclusion criteria were refusal to provide informed consent; maternal age < 18 or >45 years; BMI < 18.5 or ≥30.0 kg/m^2^; multiple pregnancy; severe chronic somatic disease; chronic arterial hypertension; use of antihypertensive drugs or statins; thyroid dysfunction (hyper- or hypothyroidism); allergic reactions to proposed therapeutic agents; and anemia.

Participants (women aged 20–37 years with a mean age of 27.6 ± 2.4 years) were residents of the Ternopil region, western Ukraine (48–50° N). No significant differences were observed between groups in maternal age, BMI, erythrocyte count, or hemoglobin level (*p* > 0.05).

### 4.2. Histology

For histological examination, pieces of the placenta were fixed overnight in 10% neutral buffered formalin. The tissue was processed in a histoprocessor LOGOSone (Milestone, Sorisole, Italy). Placenta paraffin sections were stained with Hematoxylin and Eosin and evaluated using a Nikon Eclipse Ci-E light microscope (Nikon, Tokyo, Japan), and images were captured with a digital camera (Sigeta M3CMOS 14000, Kyiv, Ukraine).

A total of 650 microscopic fields of view (FOV) were analyzed from histological slides at 10× magnification using a Nikon Eclipse Ci-E microscope to determine the percentages of histopathological findings in placental sections. The presence or absence of microscopic findings—including infarction in the decidua basalis, fibrinoid necrosis, calcifications, fibrosis of the vessel wall in chorionic villi, vascular thrombosis, villous edema, chorangiosis, and inflammation—was recorded. Findings were expressed as the percentage of positive fields of view relative to the total number examined within each group. No comparative statistical analyses were performed; data are presented descriptively to illustrate the frequency of observed lesions.

### 4.3. Immunohistochemistry

Immunohistochemistry was performed on thin formalin paraffin-embedded sections of the human placenta. The sections were stained either with a rabbit polyclonal antibody against Placental Growth Factor (PlGF) (Cat. No. ab217001, Abcam, Waltham, MA, USA), with a mouse monoclonal antibody against Vascular Endothelial Growth Factor A (VEGF A) (Cat. No. ab1316, Abcam, Waltham, MA, USA), with a rabbit polyclonal antibody against Sclerostin (Cat. No. ab264040, Abcam, Waltham, MA, USA), with a mouse monoclonal antibody against Annexin A2 (AnxA2) (Cat. No. 66035-1-Ig, Proteintech, Chicago, IL, USA). Detection was performed using a Mouse/Rabbit PolyVue Plus HRP/DAB Detection System (Diagnostic Biosystems, Pleasanton, CA, USA). The sections were then counterstained with Hematoxylin M (Biognost, Zagreb, Croatia) and mounted under coverslips.

Staining intensity in immunohistochemistry was graded on a three-tier scale: weak, moderate, and strong.

### 4.4. Electron Microscopy

For electron microscopy, fragments of the placentas were fixed in 2.5% glutaraldehyde solution (SPI supplies, West Chester, PA, USA). Post-fixation was performed using 1% osmium tetroxide solution, followed by dehydration in increasing ethanol concentration and embedded in a mixture of epoxy resins. Semithin sections (1 μm-thick) and ultrathin sections (0.4–0.6 μm-thick) were cut using an ultramicrotome Ultrotome LKB 4801 A (LKB Produkter AB, Stockholm, Sweden). Semithin sections were stained with toluidine blue and examined using a Nikon Eclipse Ci-E light microscope (Nikon, Japan). Ultrathin sections were contrasted using the Reynolds method and examined with an electron microscope PEM-125K (Electron, Sumy, Ukraine).

### 4.5. Statistical Analysis

To analyse the data of involved women, statistical analysis was performed using the statistical software package STATISTICA 6.0 for Windows (StatSoft, Tulsa, OK, USA) and Microsoft Excel (Microsoft Corporation, Redmond, WA, USA). Data were analysed as Mean ± SD (for variables with normal distribution) and Median and interquartile range (for variables with nonnormal distribution). Comparisons of numerical values were done using parametric (ANOVA, *t*-test) and nonparametric (Kruskal–Wallis, Mann–Whitney) tests. Chi-square test was used to compare frequency tables. The Spearman correlation test was used to define the relationship between values. Statistical significance was assumed at *p*-value < 0.05.

## 5. Conclusions

Placental changes in SARS-CoV-2–affected pregnancies are nonspecific and mainly related to impaired blood circulation. The most common findings include endothelial dysfunction with thrombosis, chorangiosis, villous edema, and fibrinoid necrosis, as well as the increased expression of sclerostin and Annexin A2. Thrombotic and sclerotic alterations were predominantly observed in the maternal portion of the placenta, which may impair fetal development and lead to growth restriction, miscarriage, or stillbirth. Pathological changes were most pronounced in the third trimester, with ultrastructural evidence of villous stromal edema and fibrous processes, resulting in significant disruptions to the ultrastructure of the constituent components of the fetoplacental barrier. Therefore, the manifestation of pathological changes in the placenta of COVID-19-affected women depends on the pregnancy period during which infection by SARS-CoV-2 has occurred.

The established findings in the placenta of women affected with SARS-CoV-2 infection may provide insight into the connection between COVID-19 in pregnancy and antenatal and perinatal outcomes.

## Figures and Tables

**Table 1:** Groups of study.

Group	Characteristics	Number of Patients
Control	pregnant COVID-19-negative women with a physiological course of pregnancy	10
Group I	patients who were diagnosed with COVID-19 during the 1st trimester of pregnancy; miscarriage	10
Group II	patients who were diagnosed with COVID-19 during the 2nd trimester of pregnancy	15
Group III	patients who were diagnosed with COVID-19 during the 3rd trimester of pregnancy	15

## Data Availability

The datasets and materials used and/or analyzed during the current study are available from the corresponding author upon reasonable request.
